# Potential effects of cannabinoids on audiovestibular function: A narrative review

**DOI:** 10.3389/fphar.2022.1010296

**Published:** 2022-12-20

**Authors:** Joaquin Guerra, Vinogran Naidoo, Ramon Cacabelos

**Affiliations:** ^1^ Neuro-Otolaryngology Unit, EuroEspes Biomedical Research Center, Institute of Medical Science and Genomic Medicine, Bergondo, Corunna, Spain; ^2^ Department of Neuroscience, International Center of Neuroscience and Genomic Medicine, EuroEspes Biomedical Research Center, Bergondo, Corunna, Spain; ^3^ Genomic Medicine, EuroEspes Biomedical Research Center, Institute of Medical Science and Genomic Medicine, Bergondo, Corunna, Spain

**Keywords:** hearing, vertigo, cannabinoids, cannabis, THC-tetrahydrocannabinol, CBD-cannabidiol

## Abstract

The growing interest in the development of drugs that target the endocannabinoid system has extended to conditions that affect the audiovestibular pathway. The expression of cannabinoid (CB) receptors in that pathway has been widely demonstrated, indicating a therapeutic potential for drug development at this level. These medications may be beneficial for conditions such as noise-induced hearing loss, ototoxicity, or various forms of vertigo of central or peripheral origin. The therapeutic targets of interest include natural or synthetic compounds that act as CB1/CB2 receptor agonists/antagonists, and inhibitors of the endocannabinoid-degrading enzymes FAAH and MAGL. Furthermore, genetic variations implicated in the response to treatment and the development of related disorders such as epilepsy or migraine have been identified. Direct methods of administering these medications should be examined beyond the systemic strategy.

## 1 Introduction

Auditory and vestibular disorders comprise a heterogeneous group of conditions that have different pathophysiological mechanisms. Depending on the disease, they may affect different structures of the inner ear, cochleovestibular nerve, central signaling or processing nuclei. At the same time, these disorders can either increase or decrease sensory and neural excitability. For example, increased sensory and neural excitability occurs during an acute vestibular crisis (e.g., vestibular neuritis, migraine, and labyrinthitis) or during tinnitus. By contrast, in other disorders such as chronic vestibular hypofunction or age-related hearing loss, there is a deterioration of sensory and neural activity (Peelle and Wingfield, 2016). The neurochemistry of these audiovestibular disorders includes diverse neurotransmitters with excitatory (glutamate, acetylcholine), inhibitory (GABA, glycine), modulatory (histamine, noradrenaline) and other actions (enkephalin, motilin, somatostatin, dopamine and serotonin) (Guerra and Cacabelos, 2019). As a result, and depending on the case, therapeutic approaches differ, with depressive drugs administered in cases of increased neuronal excitability, and stimulants prescribed in cases of decreased neuronal activity.

Regardless of their etiology, acute vestibular disorders are treated with vestibular sedatives. Most of the medications used to treat audiovestibular syndromes are also used to treat central nervous system (CNS) disorders. Anticholinergics, antihistamines, benzodiazepines, calcium-channel blockers and dopamine receptor antagonists minimize nystagmus and vegetative symptoms that include tachycardia, sweating, nausea, and vomiting. To avoid delaying vestibular compensation, prescribing those drugs that have sedative effects should be used in the short term (Hain and Uddin, 2003). Drugs that decrease oxidative stress and inflammation and increase cochlear vascularization can be used to slow hearing loss. Antioxidants, neurotrophic factors, calcium-channel blockers, vasodilators, antiglutamatergic, steroid, and other anti-inflammatory and antiapoptotic agents are also commonly used to treat auditory disorders. However, the majority of these drugs are only protective and there is weak evidence of their efficacy (Sha and Schacht, 2016).

Knowledge of the patient’s pharmacogenetic profile is essential for reducing the risk of adverse effects and for optimizing treatment response; this prescription of pharmacogenetic profile-based strategies improves the therapeutic response. Single nucleotide polymorphisms (SNPs) in genes encoding drug-metabolizing enzymes, drug transporters and drug targets are primarily responsible for the variations in individual drug response. One of the potential consequences of using pharmacogenomics in clinical trials and molecular therapeutics is that a given disease may be treated based on genomic and biological markers, selecting drugs optimized for individual patients or for groups of patients with similar genomic profiles. This aspect has been little explored in the scientific literature (Guerra and Cacabelos, 2019).

The endocannabinoid (EC) system is distributed widely in the brain and is also expressed in the audiovestibular pathway (Gong et al., 2006; Chi and Kandler, 2012; Trattner, Berner, Grothe and Kunz, 2013). There has been increased interest in the development of drugs that act either as an agonist or antagonist on the EC system. This review summarizes current understanding on the role of the EC system for the future use and development of natural- or synthetically-derived compounds that act on the audiovestibular pathway. To pursue those objectives, it is necessary to fully understand the properties of the EC system and the pharmacogenetic profiles of drugs that act on the EC system.

## 2 The endocannabinoid system

CB1 (encoded by CNR1 gene, locus 6q15) and CB2 (encoded by CNR2 gene, locus 1p36.11) are the 2 G protein-coupled cannabinoid (CB) receptors. Activation of the Gi/Goα subunits reduces intracellular cAMP and increases MAPK levels. CB1 can occasionally activate the G-protein Gs thereby enhancing cAMP levels and the CB2 Gβγ subunits thus activating the MAPK-ERK signaling pathway. Most of the CB receptors in the CNS are CB1 receptors whose function is primarily neuromodulatory through inhibiting GABAergic and glutamatergic neurotransmission. However, CB2 receptors are functionally anti-inflammatory. Other clinically cannabinoid orphan receptors of interest in this review are GPR18 (13q32.3), GPR55 (2q37.1 locus) and GPR119 (Xq26.1 locus), that also encode G protein-coupled receptors (Lu and Mackie, 2016; Zou and Kumar, 2018; Kilaru and Chapman, 2020). Regarding endogenous cannabinoids (CBs), six ligands that act as neurotransmitters have been identified: arachidonoylethanolamine (anandamide/AEA), 2-arachidonoylglycerol (2-AG), 2-arachidonoylglyceryl ether (noladin ether) N-arachidonoyl-dopamine (NADA), Virodhamine, and lysophosphatidylinositol (LPI) (Gallelli et al., 2018). Some of these molecules have anti-inflammatory, sedative, or analgesic properties that are of interest in several audiovestibular disorders, with AEA and 2-AG being the most clinically relevant (Zogopoulos, Vasileiou, Patsouris and Theocharis, 2013). Fatty acid amide hydrolase (FAAH, encoded by FAAH gene, locus 1p33) and monoacylglycerol lipase (MAGL, encoded by MGLL gene, locus 3q21.3) are the enzymes responsible for the degradation of the endocannabinoids (Lu and Mackie, 2016; Zou and Kumar, 2018; Kilaru and Chapman, 2020).

## 3 Cannabinoids: Targets, effects and pharmacogenomics

More than one hundred CBs derived from the *Cannabis sativa* plant have been identified. Of these, tetrahydrocannabinol (THC), cannabidiol (CBD) and cannabinol (CBN) are the most studied and for which the most clinical data are available (Chandra et al., 2020).

In addition to the commercialization of cannabinoid-containing medications and supplements, several clinical studies are currently investigating the effects of 1) synthetic cannabinoid-derived drugs (CB1/CB2 receptor agonists/antagonists), and 2) non-cannabinoid-derived compounds (MAGL/FAAH inhibitors) that belong to a part of a different class of biologically active molecules, that regulate the EC system. These compounds have been used in clinical trials and observational studies to manage treatment-resistant epilepsy (Devinsky et al., 2016), ALS (amyotrophic lateral sclerosis) (Weber et al., 2010), MS (multiple sclerosis) (Collin, Davies, Mutiboko and Ratcliffe, 2007; Zajicek et al., 2012), Alzheimer’s disease (Herrmann et al., 2019), schizophrenia (Leweke et al., 2012; McGuire et al., 2018), acute and chronic pain (van de Donk et al., 2018; Weizman et al., 2018), and several other conditions (Kogan and Mechoulam, 2007; Perin et al., 2020; Khalsa et al., 2022). Since many of the above-mentioned and other nervous system disorders also affect the auditory and vestibular pathway, and are associated with dizziness, nystagmus and auditory symptoms, these molecules may help address several types of audiovestibular disorders. Moreover, symptoms that show these neurological diseases appear in peripheral audiovestibular pathology, such as nystagmus, tinnitus or hearing loss (Baguley et al., 2013; Zwergal and Dieterich, 2020).

THC and its synthetic derivative dronabinol are CB1/CB2 receptor agonists. They mimick the effects of ECs in the CNS and modulate the excitatory effect of other neurotransmitters including glutamate. These agents may adversely enhance the tachycardic response of anticholinergics and sympathomimetics and can cause tinnitus, ataxia, and seizures as side effects. Dronabinol’s half-life is 25–36 h, while its metabolites can last up to 59 h. Nabilone, another synthetic cannabinoid that mimics THC, has a more variable half-life, ranging from 2 to 35 h (Cacabelos, 2012). The analogous use of THC and its synthetic derived drugs with opioid derived drugs, antidepressants and anticonvulsants requires a dose reduction due to greater analgesic efficacy and/or higher drug toxicity caused by down-regulation of transporter efflux or enzymatic inhibition (Vázquez et al., 2020). The pharmacogenetic profile of cannabinoid derivatives is still under development, especially with regard to new synthetic derivatives. Dronabinol’s mechanistic genes are *CNR1* and *CNR2*, and it acts as an agonist. Its metabolic genes are *CYP3A4, CYP2C9, CYP2C19, CYP1A2, CYP2B6, CYP2D6, CYP1A1, CYP2J2, CYP3A5, CYP3A7, CYP1B1, CYP2A6, CES1* and *PTGS1*, and the transporter genes include *ABCG2, ABCB1* and *ABCC1*. Nabilone displays the same mechanistic genes as Dronabinol, but it binds partially to its receptors; *CYP2C9, CYP3A4, CYP2C8, CYP2E1* and *CYP2J2* are its metabolic genes.

Cannabidiol, however, is a non-competitive CB1/CB2 receptor antagonist. It reduces neuronal hyperexcitability by modulating Ca^2+^ entry *via* GPR55 and TRPV1 receptors, and by inhibiting ENT-1-receptor-mediated adenosine reuptake (Ibeas Bih et al., 2015). It has a half-life of 18–32 h and causes somnolence, sedation, diarrhea, and changes in appetite as its side effects. It may reduce the adverse effects caused by THC and its derivatives. Drug-drug interactions include regular medications such as anticonvulsants, statins or common analgesics (Vázquez et al., 2020; Davis et al., 2021). The mechanistic genes of cannabidiol are much broader, with diverse targets, as seen in Table 1. *CYP3A4, CYP2C19, CYP1A2, CYP2B6, CYP2C9, UGT1A7, UGT1A9, UGT2B7, UGT2B17, CYP1A1, CYP1B1, CYP2C8, CYP2D6, CYP2E1, CYP3A5, CYP2A6, CYP3A7, CYP2J2, CES1, AANAT, FAAH, ALOX5, ALOX15*, and *CRYZ* are its metabolic genes. Transporter genes associated with cannabidiol include *ABCC1, ABCG2, SLC29A1*, and *ABCB1* (Cacabelos, 2012; Wishart et al., 2017).

**TABLE 1:** Profile of the different cannabinoids and drugs that act on the cannabinoid system that may be effective in the treatment of audiovestibular disorders.

Drug and properties		Targets	
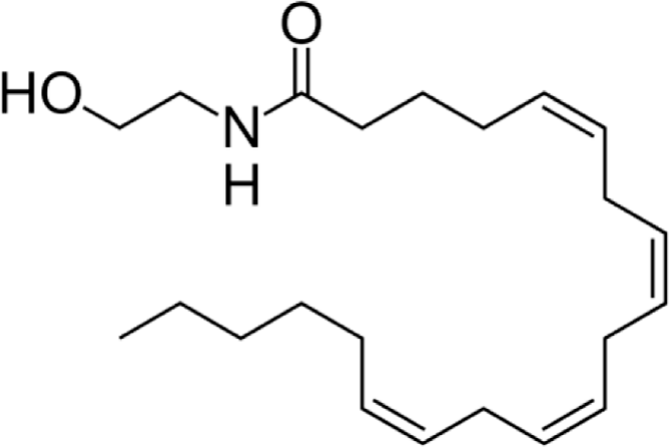	**Name:** Anandamide **IUPAC name:** (5Z,8Z,11Z,14Z)-N-(2-hydroxyethyl)icosa-5,8,11,14-tetraenamide **Molecular mass:** 347.53 g·mol−1 **Molecular formula:** C_22_H_37_NO_2_ **Category:** Endocannabinoids	**Agonists** CB1; TRPV1	
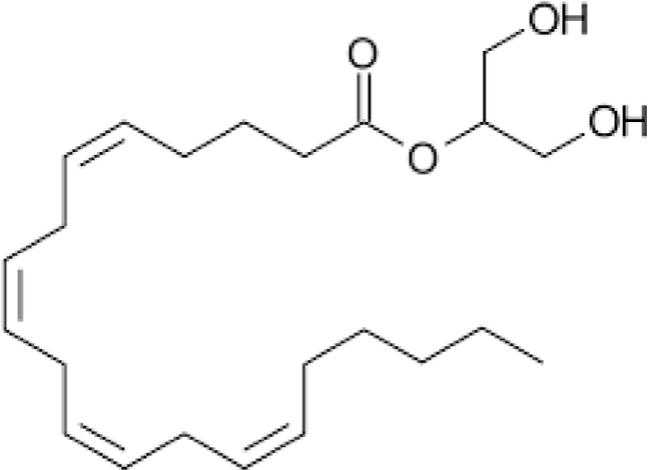	**Name:** 2-Arachidonoylglycerol (2-AG) **IUPAC name:** 1,3-dihydroxypropan-2-yl (5Z,8Z,11Z,14Z)-icosa-5,8,11,14-tetraenoate **Molecular mass:** 378.3 g·mol−1 **Molecular formula:** C_23_H_38_O_4_ **Category:** Endocannabinoids	**Agonists** CB1; CB2	
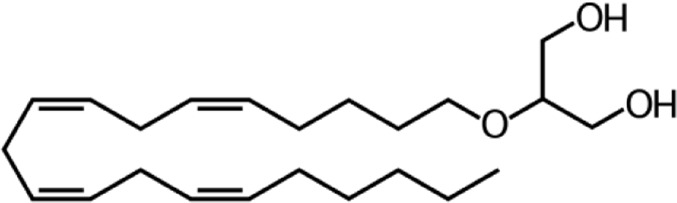	**Name:** 2-Arachidonyl glyceryl ether (2-AGE; Noladin ether) **IUPAC name:** 2-[(5Z,8Z,11Z,14Z)-icosa-5,8,11,14-tetraenoxy]propane-1,3-diol **Category:** Endocannabinoids **Molecular mass:** 364.56 g·mol−1 **Molecular formula:** C_23_H_40_O_3_	**Agonists** CB1; CB2 (*weak*)	
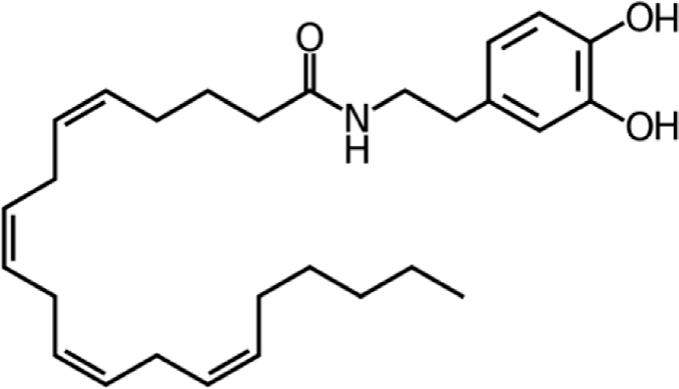	**Name:** N-Arachidonoyl dopamine (NADA) **IUPAC name:** (5Z,8Z,11Z,14Z)-N-[2-(3,4-dihydroxyphenyl)ethyl]icosa-5,8,11,14-tetraenamide **Molecular mass:** 439.63 g·mol−1 **Molecular formula:** C_28_H_41_NO_3_ **Category:** Endocannabinoids	**Agonists** CB1; TRPV1	
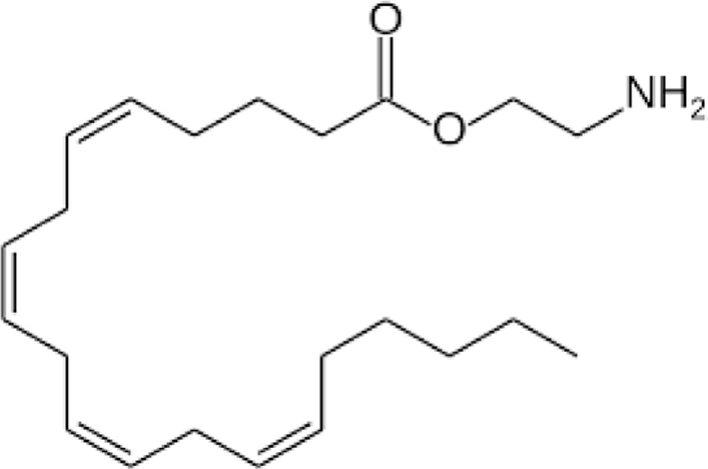	**Name:** Virodhamine (O-arachidonoyl ethanolamine; O-AEA) **IUPAC name:** 2-aminoethyl (5Z,8Z,11Z,14Z)-icosa-5,8,11,14-tetraenoate **Molecular mass:** 347.53 g·mol−1 **Molecular formula:** C_22_H_37_NO_2_ **Category:** Endocannabinoids	**Agonists** CB2	**Antagonists** CB1
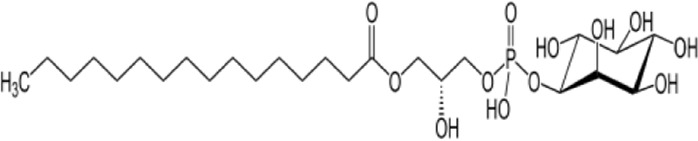	**Name:** Lysophosphatidylinositol (LPI, lysoPI) **IUPAC name:** [(2S)-2-hydroxy-3-[hydroxy-[(2R,3S,5R,6R)-2,3,4,5,6-pentahydroxycyclohexyl]oxyphosphoryl]oxypropyl] acetate **Molar mass:** 572.629 g·mol**−**1 **Molecular formula:** C_25_H_49_O_12_P **Category:** Endocannabinoids	**Agonists** GPR55	
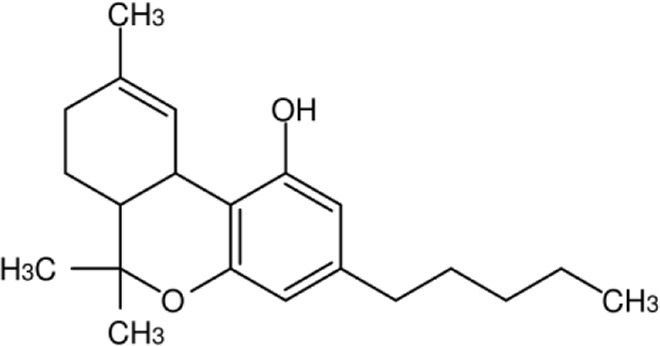	**Name:** THC/Dronabinol **IUPAC name:** (6aR,10aR)-6,6,9-trimethyl-3-pentyl-6a,7,8,10a-tetrahydrobenzo [c]chromen-1-ol **Molar mass:** 314.469 g·mol−1 **Molecular formula:** C_21_H_30_O_2_ **Category:** Phytocannabinoids/Synthetic cannabinoids	**Agonists** CB1; CB2	
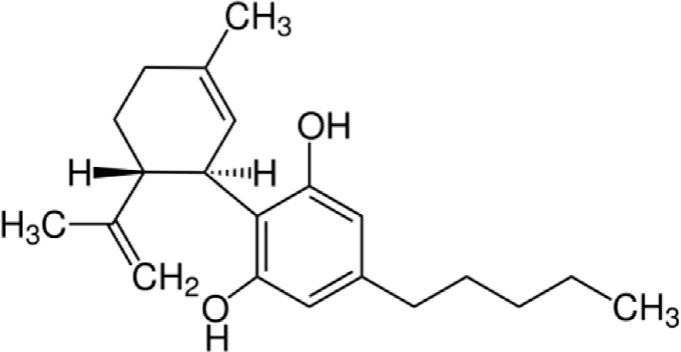	**Name:** Cannabidiol **IUPAC name:** 2-[(1R,6R)-3-methyl-6-prop-1-en-2-ylcyclohex-2-en-1-yl]-5-pentylbenzene-1,3-diol **Molar mass:** 314.469 g·mol−1 **Molecular formula:** C_21_H_30_O_2_ **Category:** Phytocannabinoids	**Agonists** HTR1A; HTR2A; TRPA1	**Activators** PPARG; TRPV1; TRPV2; TRPV3; TRPV4; ADORA1
		**Inverse agonists** GPR12	
			**Inhibitors** PTGS1; PTGS2; ACAT1; IDO1; NQO1; CAT; SOD; AANAT; NAAA
		**Antagonists** CB1; CB2; GPR55; HTR3A	
			**Stimulators** HMGCR; GSR; GSR; GPX
		**Modulators** CB1	
			**Other targets** GLRA1; GPR18; CHRNA7; OPRD1; OPRM1; CACNA1G CACNA1H; CACNA1I; TRPM8; VDAC1
		**Potentiators** GLRA3	
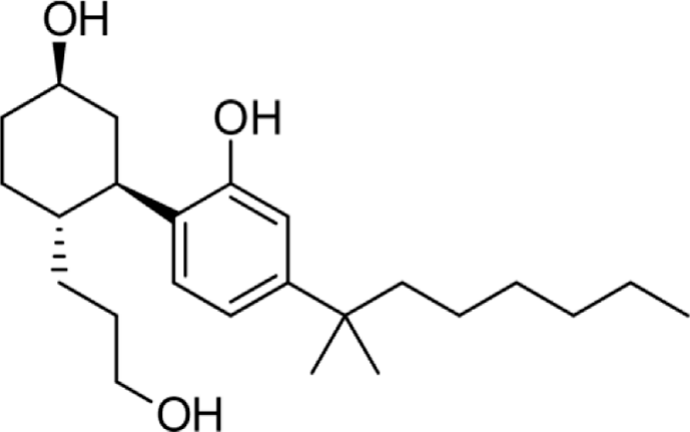	**Name:** CP 55,940 **IUPAC name:** 2-[(1R,2R,5R)-5-hydroxy-2-(3-hydroxypropyl)cyclohexyl]-5-(2-methyloctan-2-yl)phenol **Molar mass:** 376.581 g·mol−1 **Molecular formula:** C_24_H_40_O_3_ **Category:** Synthetic cannabinoids	**Agonists** CB1; CB2; HTR5A	**Antagonists** GPR55
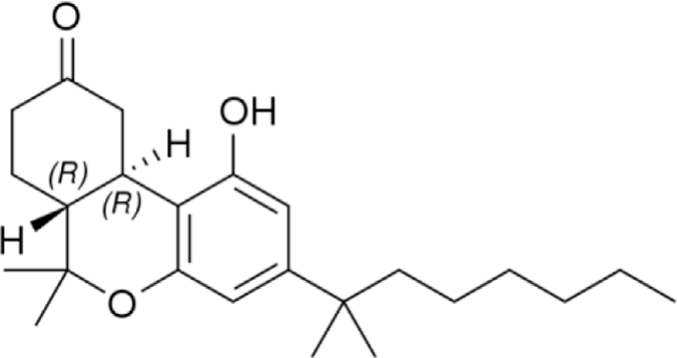	**Name:** Nabilone **IUPAC name:** (6aR,10aR)-1-hydroxy-6,6-dimethyl-3-(2-methyloctan-2-yl)-7,8,10,10a-tetrahydro-6aH-benzo [c]chromen-9-one **Molar mass:** 372.549 g·mol−1 **Molecular formula:** C_24_H_36_O_3_ **Category:** Synthetic cannabinoids	**Agonists** CB1 (*partial*); CB2 (*partial*)	
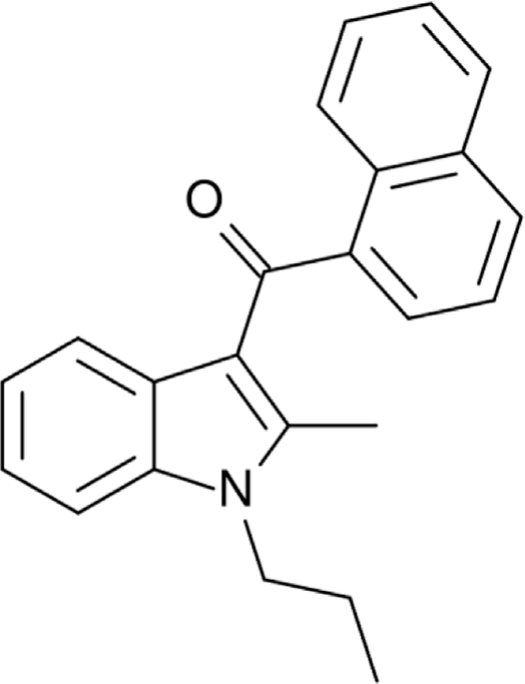	**Name:** JWH-015 **IUPAC name:** (2-methyl-1-propylindol-3-yl)-naphthalen-1-ylmethanone **Molar mass:** 327.427 g·mol−1 **Molecular formula:** C_23_H_21_NO **Category:** Synthetic cannabinoids	**Agonists** CB2; CB1 (*weak*)	
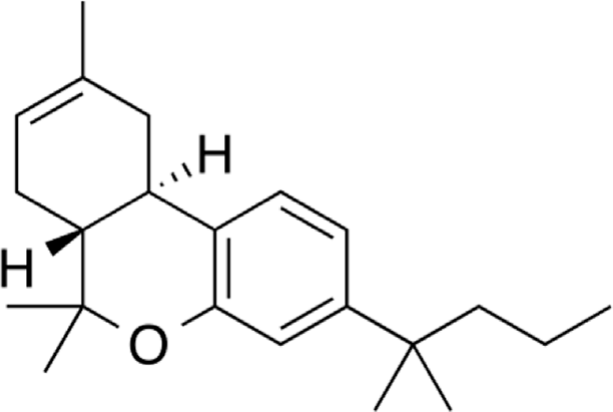	**Name:** Dimethylbutyl-deoxy-Delta-8-THC (JWH-133) **IUPAC name:** (6aR,10aR)-6,6,9-trimethyl-3-(2-methylpentan-2-yl)-6a,7,10,10a-tetrahydrobenzo [c]chromene **Molar mass:** 312.497 g·mol−1 **Molecular formula:** C_22_H_32_O **Category:** Synthetic cannabinoids	**Agonists** CB2	
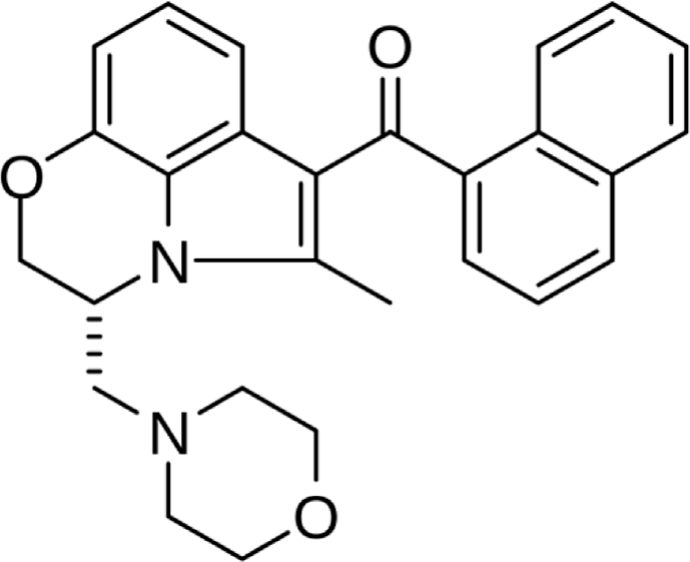	**Name:** WIN55,212–2 **IUPAC name:** [(11R)-2-methyl-11-(morpholin-4-ylmethyl)-9-oxa-1-azatricyclo [6.3.1.04,12]dodeca-2,4 (12),5,7-tetraen-3-yl]-naphthalen-1-ylmethanone **Molar mass:** 338.407 g•mol−1 **Molecular formula:** C20H22N2O3 **Category:** Synthetic cannabinoids	**Agonists** CB1; CB2; PPARα; PARγ	
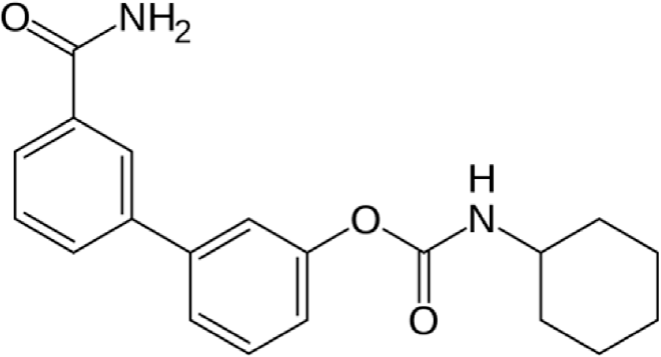	**Name:** URB597 (KDS-4103) **IUPAC name:** [3-(3-carbamoylphenyl)phenyl] N-cyclohexylcarbamate **Molar mass:** 338.407 g•mol−1 **Molecular formula:** C_20_H_22_N_2_O_3_ **Category:** Endocannabinoid reuptake inhibitor	**Inhibitor** FAAH	
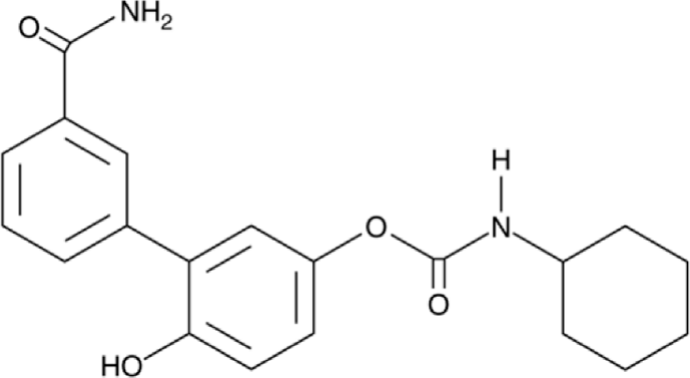	**Name:** URB937 **IUPAC name:** N-cyclohexyl-carbamic acid, 3'-(aminocarbonyl)-6-hydroxy [1,1′-biphenyl]-3-yl ester **Molar mass:** 338.407 g•mol−1 **Molecular formula:** C_20_H_22_N_2_O_4_ **Category:** Endocannabinoid reuptake inhibitor	**Inhibitor** FAAH	
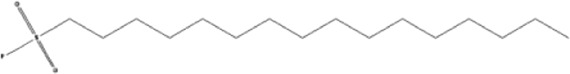	**Name:** AM374 **IUPAC name:** Hexadecanesulfonyl fluoride **Molar mass:** 308.219 g•mol−1 **Molecular formula:** C_16_H_33_FO_2_S **Category:** Endocannabinoid reuptake inhibitor	**Inhibitor** FAAH	
		**Other targets** PPT1	
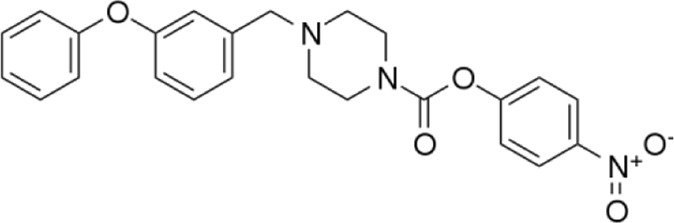	**Name:** JZL195 **IUPAC name:** 4-nitrophenyl 4-(3-phenoxybenzyl)piperazine-1-carboxylate **Molar mass:** 433.464 g•mol−1 **Molecular formula:** C_24_H_23_N_3_O_5_ **Category:** Endocannabinoid reuptake inhibitor	**Inhibitor** FAAH; MAGL	
**Name:** Dietressa^©^ **Category:** Synthetic cannabinoid antibodies		**Antagonists** CB1	
**Name:** Brizantin^©^ **Category:** Synthetic cannabinoid antibodies		**Antagonists** CB1	

*CNR1* and *CNR2* variants show mixed results in terms of cannabis dependency. *CNR1*, -64 + 1046T>G and c.-63-9c.1359G>A variants show an association with drug dependency, while 597T>C -64 + 1046T>G c.*3475A>G are also linked to drug dependency in single studies, although with no replication of results. The *CNR2* 188A>G and 946C>T variants alter its CB2 receptor function in human embryonic kidney-293 cells. Because the *COMT* rs4680 472A>G Val158Met variant is linked to memory impairment, alterations in executive function, and psychosis, the use of cannabis derivatives should be approached with caution with corresponding adjustments in prescribed doses. The *FAAH* 385C>APro129Thr variant may be involved in excessive consumption of cannabis, although other authors did not find any association between FAAH variants. The *ABCB1* rs10456423455C>T Ile1145Ile variant is associated with cannabis dependency (Hryhorowicz et al., 2018).

CB1 antagonists such as rimonabant, surinabant and other diarylpyrazole derivatives have been experimentally and clinically evaluated for treating conditions unrelated to audiovestibular symptoms such as obesity or addiction disorders. However, their use has raised safety concerns due to CNS or cardiac side effects (Christopoulou and Kiortsis, 2011; Klumpers et al., 2013). Therefore, its role in treating the conditions described in this review is not defined.

Unfortunately, little is known regarding the half-life, adverse effects and pharmacogenetic profiles of other drugs of interest described in this review. One of the primary reasons is that those drugs have been used solely for research purposes. These include CB1/CB2 agonists (CP 55,940, JWH-015 and WIN55,212–2) CB1 agonists (JWH-133), CB1 antagonists (Dietressa^©^ and Brizantin^©^), FAAH inhibitors (URB597, URB937, and AM374) and FAAH/MAGL inhibitors (JZL195). The targets of these compounds are listed in Table 1 (Cacabelos, 2012; Wishart et al., 2017).

## 4 The cannabinoid system and the vestibular pathway

The association of the EC system in the vestibular pathway has been previously demonstrated by early reports that indicated that the audiovestibular pathway expresses CB1 receptors. The density of these receptors in this pathway was initially reported as modest, but functionally significant (Newsham-West et al., 1998). Furthermore, CB2 receptors are expressed in the vestibular and cochlear nuclei (Gong et al., 2006; Baek et al., 2008), with lower distribution and relevance than CB1 receptors. The EC system interacts with the different vestibular pathways such as the vestibulocerebellar tract (Suárez et al., 2008). Optogenetically-induced plasticity and motor adaptation in the cerebellum are dependent on EC signaling. When vestibular stimuli are paired with relatively small amplitude Purkinje cell calcium responses, this Purkinje cell activation induces decreased vestibulo-ocular reflex gain. By contrast, pairing with large magnitude calcium responses promotes vestibulo-ocular reflex gain. This indicates that EC signaling acts downstream of Purkinje cell calcium elevation (Bonnan et al., 2021). CB1 receptors modulate the vestibular reflex, as indicated by their activation following unilateral vestibular deafferentiation in rats. However, their expression does not change during vestibular compensation (Ashton et al., 2004). In rats, bilateral vestibular deafferentation causes lifelong spatial memory impairments and hippocampal dysfunction (Baek et al., 2010), that is associated with CB1 receptor down-regulation in the CA3 area of the rat hippocampus (Baek et al., 2011).

### 4.1 Cannabinoids and vestibular disorders

Despite the abundance of data indicating the presence and participation of the EC system in the vestibular pathway, there are currently only a few publications (Schon et al., 1999; Pradeep et al., 2008) and just one clinical trial (Barchukov et al., 2015), which limits their clinical impact.

In animal models, the administration of the potent non-selective CB receptor agonist CP-55,940 affects the vestibular pathway, comparable to the action of certain metabotropic glutamate receptor agonists, generating dizziness. These effects are sustained and involve neurons in the medial vestibular nucleus (Newsham-West et al., 1998). TRPV1 pathways, which are targets of CBD, are associated with motion sickness processes as observed in Trpv1-null mice (Inprasit et al., 2018). Anecdotal case-reports indicate a reduction or suppression of congenital, and pendular nystagmus associated with multiple sclerosis in cannabis users (Schon et al., 1999; Pradeep et al., 2008). However, the use of nabilone has no effect on nystagmus, indicating that the use of THC agonists alone may not be sufficient to suppress it (Schon et al., 1999). The inhibitory action of CB agonists, and other compounds found in *Cannabis Sativa*, may suppress nystagmus; however, the lack of evidence pointing to the dosages used in the case reports limits these conclusions (Schon et al., 1999; Pradeep et al., 2008). A clinical trial evaluated the role of active-release preparations containing CB receptor antagonists (marketed in Russia under the Dietressa^©^ and Brizantin^©^ brands); these preparations incorporated antibodies against the CB1 receptor. In that study, patients were evaluated over 1 h after the test dose (1 tablet) of the preparation/placebo. In the follow-up period, the preparation/placebo (1 tablet) was given four times at 30-min intervals. The tolerance to accelerations in a model of vertigo with healthy subjects was increased, with no change in nystagmus duration or recovery time (Barchukov et al., 2015). In theory, cannabinoid antagonists may be advantageous during vestibular compensation. However, there are concerns about these products, related to the utilization of ultra-low doses of the active substance and limited information regarding their molecular properties.

Potential uses of CBs for other symptoms associated with vestibular disorders are described below.

#### 4.1.1 Seizures

Vestibular epilepsy is a rare condition that affects areas in the temporal cortex responsible for motor coordination. Its key characteristics are the presence of rapidly-discharging EEG activity and a favorable response to antiepileptic therapy (Hewett and Bartolomei, 2013). On the other hand, several anticonvulsants including diazepam, gabapentin and carbamazepine are used to treat several vestibular disorder subtypes, even those unrelated to seizures (Casani et al., 2021). CBD is a proposed drug indicated for treating epilepsy. Its brand-labelled product (Epidyolex^©^) is currently used to treat severe resistant epilepsies such as severe myoclonic epilepsy of infancy (Dravet syndrome; SCN1A gene, locus 2q24.3) (Scheffer et al., 2021). The anti-epileptic action of CBD can be explained by: 1) generating anti-glutamatergic activity and lowering neuronal excitability *via* GPR55 receptor antagonism and adenosine tone control; 2) activating 5HT1A receptors; and 3) modifying intracellular calcium levels. Other CB1 receptor agonists (WIN55,212–2) also have an anticonvulsant effect, similar to the anti-epileptic action of CBD (Colangeli et al., 2017). Inhibiting EC degradation has also been considered as a potential therapy for epilepsy. FAAH inhibitors (URB597, AM374) suppress hippocampal discharges and improve short- and long-term cognitive performance in rats by increasing blood-brain levels of anandamide (Karanian et al., 2007; Colangeli et al., 2017). Long-term usage of compounds (e.g., palmitoylethanolamide) that target the atypical CB receptors GPR55 and GPR11 has neuroprotective and neuromodulatory advantages in preventing epileptic convulsions in mice when combined with FAAH inhibitors (Post et al., 2018). Several antiepileptic drugs, however, are used to treat vestibular problems such as vestibular paroxysmia or central nystagmus (Strupp et al., 2011). Non-etheless, administering CBD or other CBs in these cases remains to be evaluated (Morano et al., 2020). Variants in the genes that encode aldehyde oxygenase (AOX1 rs6729738 CC) and diamine oxidase (ABP1 rs12539) increase the likelihood of an antiepileptic response. Since CBD produces an antioxidant effect, this could explain that finding. By contrast, SLC15A1 rs1339067 TT carriers exhibit a decreased antiepileptic response, indicating that this transporter limits CBD activity. EC receptor GPR18 expression in white matter is reduced by rs1339067, and hippocampal HTR3E serotonergic receptor expression is decreased by rs3749442, thus modulating the drug response in treatment-resistant epilepsy (Davis et al., 2021).

#### 4.1.2 Headache

Many patients with migraine describe neuro-otological symptoms such as phonophobia and vertigo. Vestibular migraine is a distinct entity whose diagnostic criteria include at least five episodes of migraine with/without aura and vestibular symptoms of moderate or severe intensity, lasting between 5 min and 72 h. In addition, at least half of the episodes must be associated with at least one of the following three migrainous features: 1) headache with at least two of the following four characteristics: 1) unilateral location; 2) pulsating quality; 3) moderate or severe intensity; 4) aggravation by routine physical activity; 2) photophobia and phonophobia; 3) visual aura; and 3) not better accounted for by another vestibular disorder (Lempert et al., 2012). Cannabis and its derivatives are efficacious for controlling pain and are viable agents for treating migraine and other headaches. The EC system acts *via* CB1 and CB2 receptors to alleviate pain, whereas pain stimulation occurs through TRPV1 receptors. MAGL and FAAH regulates EC levels; this analgesia affects the meninges and brainstem (Leimuranta et al., 2018). Central EC deficiency (as observed in the cerebrospinal fluid), specifically AEA and 2-AG, is a potential cause of migraine (Russo, 2016). Cannabis consumption reduces the intensity of migraine attacks but its long-term use produces tolerance (Cuttler, Spradlin, Cleveland and Craft, 2020). Moreover, a better prophylactic control was shown after treatment with medical cannabis, although with sparse evidence (Poudel et al., 2021). Both THC and CBD are responsible for this anti-migraine effect, but with the ideal ratio of these compounds yet to be determined (Mechtler et al., 2021). In preclinical studies, FAAH inhibitors used for the preventive control of migraine increased anandamide and palmitoylethanolamide levels, but has no effect in response to an acute migraine attack (Greco et al., 2020). The use of the FAAH inhibitor URB937 reduces hyperalgesia and c-Fos expression in the trigeminal caudal nucleus (TNC) and locus coeruleus in rodents. This response was accompanied by decreased neuronal gene expression of nitric oxide synthase, calcitonin gene-related peptide and cytokines (Greco et al., 2020; Greco et al., 2021a); using dual inhibitors of FAAH and MAGL produce similar effects (Greco et al., 2021b). Several rare, non-sense mutations in CNR1 increase the predisposition to developing migraine (Smith et al., 2017). CNR1 carriers with the HT6 haplotypic variant, for example, are most susceptible with an increased likelihood of headache together with nausea and photophobia (Juhasz et al., 2009). CNR2 and FAAH variants do not appear to be involved in migraine (Smith et al., 2017).

#### 4.1.3 Vomiting

Vomiting is one of the symptoms associated with a vestibular crisis; despite the limited data, THC and other agonists appear to be effective in decreasing chemotherapy-induced vomiting (Whiting et al., 2015). This central antiemetic action occurs *via* the activation of CB1 receptors, and possibly TRPV1 in the dorsal vagal complex (Darmani, 2010). But contrary to popular belief, one side effect of frequent cannabis use is vomiting (Castaneto et al., 2014). Moreover, the suggested best treatment for this hyperemesis, which is associated with THC and its plasma high levels (Chu and Cascella, 2022), is discontinuing THC consumption (Sorensen et al., 2016). THC may cause desensitizing, pharmacogenetic, or tolerance effects following its binding to CB1 receptors, as well as antagonizing the peripheral emetic effect *versus* the central antiemetic effect. However, its pathophysiology remains unclear (Sorensen et al., 2016). Although AEA alone does not diminish reduce vomiting in animal models, URB597 attenuates and even suppresses vomiting caused by chemotherapy and nicotine (Parker et al., 2009).

## 5 The cannabinoid system and the auditory pathway

EC signaling modulates neurotransmission within auditory circuits and contributes to their development (Chi and Kandler, 2012; Trattner, et al., 2013). CB1-receptor knockout mice have lower auditory thresholds at hearing frequencies greater than 8 kHz in their audiograms, than wild type mice. In terms of central auditory processing, these knockout mice outperform wild-type mice in identifying gaps in low-pass noise bursts (Toal et al., 2016). CB2 receptors are found in several areas of the inner ear, including the Organ of Corti, stria vascularis, spiral ligament, and spiral ganglion cells (Ghosh et al., 2018). CB2 receptor activation is involved in protecting against drug-induced hearing loss (Martín-Saldaña et al., 2016; Dhukhwa et al., 2021).

### 5.1 Cannabinoids and hearing disorders

There are few publications on auditory pathology than on the effect of cannabis on hearing disorders. Considering the use of CB agonists, in animal models, JWH-015, a CB2 agonist that is also a weak CB1 agonist, is otoprotective against cisplatin exposure. CB2 antagonists reverse this effect (Ghosh et al., 2018). Furthermore, another pharmacological agonist (JWH-133) activates CB2 receptors, produces an anti-inflammatory effect in the inner ear, and increases microcirculation following exposure to bacterial endotoxins (Weiss et al., 2021). Although these results appear promising, they were performed in animal models, and therefore the translational implications for humans are quite limited given the absence of studies in this regard.

As mentioned previously, CBD modulates the TRPV1 receptor. TRPV1 and other TRPs are up-regulated when exposed to an ototoxic agent (Kitahara et al., 2005) and can exert dual effects on hearing function, depending on which drug acts on TRP channels. For example, capsaicin would be protective while an aminoglycoside would exacerbate the inflammatory response (Ramkumar et al., 2022). TRPV1 activity begins after the activation of NOX3 NADPH oxidase and STAT1 and STAT3 transcription factors that mediate oxidative stress, which cause inflammation and cochlear apoptosis (Ramkumar et al., 2022). CBD may theoretically be an otoprotective drug, but no reports to date have demonstrated its efficacy in this regard.

Concerning tinnitus, several reviews have demonstrated a detrimental effect of cannabis and its derivatives on the development of tinnitus. Based on data obtained from animal models, null or even negative effects of CB1 receptor agonists on tinnitus have been reported in response to noise exposure and the use of ototoxic drugs (Zheng et al., 2010; Zheng et al., 2015; Berger et al., 2017). In humans, these findings have also been observed according to the results of surveys and clinical trials; paradoxically, a patient with intracranial hypertension showed tinnitus relief following dronabinol consumption (Narwani et al., 2020). Auditory excitability extends to the auditory cortex and may account for the auditory hallucinations reported in some subjects **(** Nestoros et al., 2017). It is unknown whether the use of cannabinoid antagonists may be effective for controlling tinnitus.

## 6 Conclusion

The present review shows that the EC system regulates audiovestibular function. The few but significant references to the use of CB agonists/antagonists, and MAGL/FAAH inhibitors that act on the EC system indicate that they may be effective for treating audiovestibular disorders. The neuromodulatory effects of those compounds would act on symptoms as diverse as dizziness, nystagmus, nausea, vomiting, tinnitus or headache across different vestibular syndromes of central and peripheral origin (linked or not to neurological pathology), in some cases with inhibitory effects, particularly CB agonists, with sedative action and excitatory effects in others, and with compensatory or protective functions, as is typical in the case of CB antagonists. A critical aspect concerning the use of drugs that act on the EC system is the absence and/or variability of doses used in all studies, even in those studies performed in animal models. This severely limits the application of EC system-targeting drugs and standardization in humans. A further issue that requires discussion is the lack of pharmacogenetic profiles for most cannabinoid derivatives, which affects all conditions that may benefit from their use. However, an understanding of the pharmacogenetics of the cannabinoid derivatives dronabinol, nabilone and cannabidiol is undoubtedly invaluable. Genes related to the metabolism and/or to the mechanisms of action of these drugs contribute to problems with therapeutic efficacy and safety of those agents. A pharmacogenomic strategy to optimize the use of drugs that interact with the EC system is required for successful, personalized therapy. Finally, whereas systemic drug administration allows for wider drug distribution to exert central and peripheral effects, the effects of local delivery of these agents to the inner ear *via* more invasive direct approaches are unknown. This application would be particularly appealing for those drugs with peripheral action or those with minimal CNS effects.
